# Supplementary material for: Stigma, depression, quality of life, and the need for psychosocial support among people with tuberculosis in Indonesia: A multi-site cross-sectional study
Source: PLOS Glob Public Health. 2024 Jan 8;4(1):e0002489. doi: 10.1371/journal.pgph.0002489 (PMC10773931; doi:10.1371/journal.pgph.0002489)
Supplement: S1 Appendix — (DOCX) [file pgph.0002489.s002.docx]

**S1 Appendix. Culturally adapted and Validated TB-Stigma Scale**

In the Characterising and Addressing the Psychosocial Impact of Tuberculosis in Indonesia (CAPITA) research programme, we culturally adapted and validated TB-Stigma Scale which was previously developed by Van Rie et al. to Indonesian context. During this process, there was some adjustment to item questions and deletion of an item. The final Indonesian version of TB-Stigma Scale are below.

**Form A: Patient perspectives toward tuberculosis**

***Instructions:*** *From now on, I shall read the statements which explain about how people with HIV/AIDS feel. After I read each statement, please answer whether you agree or disagree that people with HIV/AIDS in your community feel like that. If you agree, I will ask how you agree, agree or strongly agree. If you disagree, I will ask how you disagree, disagree or strongly disagree. You can refuse to answer any questions that make you feel uncomfortable.*

| **Items** | **Strongly disagree** | **Disagree** | **Agree** | **Strongly agree** |
| --- | --- | --- | --- | --- |
| 1. I feel hurt by how others react to knowing that I have TB |  |  |  |  |
| 2. I have lost friends when I shared with them that I have TB |  |  |  |  |
| 3. I feel lonely |  |  |  |  |
| 4. I am afraid of going to TB clinics because other people may see me there |  |  |  |  |
| 5. I am afraid to tell people outside my family that I have TB |  |  |  |  |
| 6. I am afraid to tell others that I have TB because others may think that I also have HIV/AIDS |  |  |  |  |
| 7. I feel guilty because my family has the burden of caring for me |  |  |  |  |
| 8. I choose carefully who I tell about having TB |  |  |  |  |
| 9. I feel guilty for getting TB because of my smoking, drinking, or other risky behaviours |  |  |  |  |
| 10. I am worried about having HIV/AIDS |  |  |  |  |
| 11. I am afraid of other people to tell my family that I have TB |  |  |  |  |

**Form B:** **Community perspectives toward tuberculosis**

***Instructions:*** *From now on, I shall read the statements, which explain about how your community feels towards people with TB. After I read each statement, please answer whether you agree or disagree that such events occur in your community. If you agree, I will ask how you agree, agree or strongly agree. If you disagree, I will ask how you disagree, disagree or strongly disagree. You can refuse to answer any questions that make you feel uncomfortable.*

| **Items** | **Strongly disagree** | **Disagree** | **Agree** | **Strongly agree** | |
| --- | --- | --- | --- | --- | --- |
| 1. Some people may not want to eat or drink with friends who have TB |  |  |  |  |  |
| 2. Some people feel uncomfortable about being near those with TB |  |  |  |  |  |
| 3. If a person has TB, some community members will behave differently towards that person for the rest of his ⁄ her life have HIV/AIDS |  |  |  |  |  |
| 4. Some people do not want those with TB playing with their children |  |  |  |  |  |
| 5. Some people keep their distance from people with TB |  |  |  |  |  |
| 6. Some people think that those with TB are disgusting |  |  |  |  |  |
| 7. Some people do not want to talk to others with TB |  |  |  |  |  |
| 8. Some people are afraid of those with TB |  |  |  |  |  |
| 9. Some people try not to touch others with TB |  |  |  |  |  |
| 10. Some people may not want to eat or drink with relatives who have TB |  |  |  |  |  |

Each set of stigma scales was assessed on a four-point likert scale which was scored with the response categories: (0) strongly disagree, (1) disagree, (2) agree, and (3) strongly agree; higher responses were related to higher stigma. TB-Stigma scores were calculated following the original tool’s guidance.

**TB-Stigma Score** = (sum of item scores x 50) / (3 x number of item).

In addition, to enhance interpretation and potential future policy dialogue, we modified the scores to according to cohort quartiles:

1. No stigma (no stigmatisation in all items)
2. Low (TB-Stigma score <16.67)
3. Moderate (16.68-33.33)
4. High TB-Stigma (>33.33).
