# Supplementary material for: Stigma, depression, quality of life, and the need for psychosocial support among people with tuberculosis in Indonesia: A multi-site cross-sectional study
Source: PLOS Glob Public Health. 2024 Jan 8;4(1):e0002489. doi: 10.1371/journal.pgph.0002489 (PMC10773931; doi:10.1371/journal.pgph.0002489)
Supplement: S2 Appendix — (DOCX) [file pgph.0002489.s003.docx]

**S2 Appendix. Patient Health Questionnaire-9 (PHQ-9)**

We used a validated Indonesian version of Patient Health Questionnare-9 (PHQ-9) to measure depression symptoms among study participants. The PHQ-9 form and interpretation guidance are shown below.

| **PATIENT HEALTH QUESTIONNAIRE-9**  **(PHQ-9)** | | | | |
| --- | --- | --- | --- | --- |
| **Over the last 2 weeks, how often have you been bothered by any of the following problems?** *(Use “*✔*” to indicate your answer)* | **Not at all** | **Several days** | **More than half the days** | **Nearly every day** |
| **1.** Little interest or pleasure in doing things | 0 | 1 | 2 | 3 |
| **2.** Feeling down, depressed, or hopeless | 0 | 1 | 2 | 3 |
| **3.** Trouble falling or staying asleep, or sleeping too much | 0 | 1 | 2 | 3 |
| **4.** Feeling tired or having little energy | 0 | 1 | 2 | 3 |
| **5.** Poor appetite or overeating | 0 | 1 | 2 | 3 |
| **6.** Feeling bad about yourself — or that you are a failure or have let yourself or your family down | 0 | 1 | 2 | 3 |
| **7.** Trouble concentrating on things, such as reading the newspaper or watching television | 0 | 1 | 2 | 3 |
| **8.** Moving or speaking so slowly that other people could have noticed? Or the opposite — being so fidgety or restless that you have been moving around a lot more than usual | 0 | 1 | 2 | 3 |
| **9.** Thoughts that you would be better off dead or of hurting yourself in some way | 0 | 1 | 2 | 3 |

**FOR OFFICE CODING**  *0*  **+** ______ **+** ______ **+** ______

**=Total Score:** ______

**Interpretation of PHQ-9 total score.**

| **Total score** | **Interpretation 1** | **Interpretation 2** |
| --- | --- | --- |
| 1–4 | Minimal depression | No to minimal depression |
| 5–9 | Mild depression | Mild to moderate depression |
| 10–14 | Moderate depression |  |
| 15–19 | Moderately severe depression | Severe depression |
| 20–27 | Severe depression |  |

**Major depression disorder (MDD)** is defined when a person indicates having more than five symptoms in PHQ9 more than half the days and Symptom 1 (Little interest or pleasure in doing things) or Symptom 2 (Feeling down, depressed, or hopeless) more than half the days.

**Other depressive disorder** is defined when a person indicates having 2-4 symptoms in PHQ9 and Symptom 1 or Symptom 2 more than half the days.
