# Supplementary material for: Stigma, depression, quality of life, and the need for psychosocial support among people with tuberculosis in Indonesia: A multi-site cross-sectional study
Source: PLOS Glob Public Health. 2024 Jan 8;4(1):e0002489. doi: 10.1371/journal.pgph.0002489 (PMC10773931; doi:10.1371/journal.pgph.0002489)
Supplement: S3 Appendix — (DOCX) [file pgph.0002489.s004.docx]

**S3 Appendix. Instrument to measure social support received and needed by people with TB.**

We developed a set of questions to assess participants’ receipt of existing social support and their perceived need for receipt of such support. The social support packages were divided into three categories: informational, emotional and instrumental supports.

1. Informational support referred to any facts, advice, or other educational materials provided to people with TB.
2. Emotional support referred to care, encouragement, and empathy showing a sense of security.
3. Instrumental support was defined as a tangible help in practical form provided through material assistance or practical program, such as counseling or meeting.

| **Social support** | **I perceive that I…** | | | | **Needs** |  |
| --- | --- | --- | --- | --- | --- | --- |
|  | **Had never  received** | **have not  received  enough** | **Have  received  enough** | **Have  received  a lot** | **Yes, I  need** | **No, I  don’t  need** |
| **Information support** | | | | | | |
| Information about TB from healthcare staff, for me | [ ] | [ ] | [ ] | [ ] | [ ] | [ ] |
| Information about TB from healthcare staff, for my family members | [ ] | [ ] | [ ] | [ ] | [ ] | [ ] |
| Information about TB from healthcare staff, for me, in peer-group meeting | [ ] | [ ] | [ ] | [ ] | [ ] | [ ] |
| **Emotional support** | | | | | | |
| Emotional support from healthcare staff | [ ] | [ ] | [ ] | [ ] | [ ] | [ ] |
| Emotional support from family members | [ ] | [ ] | [ ] | [ ] | [ ] | [ ] |
| Emotional support from peer with TB | [ ] | [ ] | [ ] | [ ] | [ ] | [ ] |
| **Instrumental support** | | | | | | |
| Home visit by healthcare staff | [ ] | [ ] | [ ] | [ ] | [ ] | [ ] |
| Peer-group meeting with people with TB | [ ] | [ ] | [ ] | [ ] | [ ] | [ ] |
| Individual psychological counseling, for me | [ ] | [ ] | [ ] | [ ] | [ ] | [ ] |
| Group counseling with other people with TB | [ ] | [ ] | [ ] | [ ] | [ ] | [ ] |
