# Supplementary material for: Stigma, depression, quality of life, and the need for psychosocial support among people with tuberculosis in Indonesia: A multi-site cross-sectional study
Source: PLOS Glob Public Health. 2024 Jan 8;4(1):e0002489. doi: 10.1371/journal.pgph.0002489 (PMC10773931; doi:10.1371/journal.pgph.0002489)
Supplement: S1 Fig — White horizontal lines in the boxplot are the median values. (DOCX) [file pgph.0002489.s005.docx]

**S1 Fig.** **Correlation between TB-Stigma and PHQ scores, and TB-Stigma score between depression groups.** White horizontal lines in the boxplot are the median values.
