# Supplementary material for: Stigma, depression, quality of life, and the need for psychosocial support among people with tuberculosis in Indonesia: A multi-site cross-sectional study
Source: PLOS Glob Public Health. 2024 Jan 8;4(1):e0002489. doi: 10.1371/journal.pgph.0002489 (PMC10773931; doi:10.1371/journal.pgph.0002489)
Supplement: S1 Table — (DOCX) [file pgph.0002489.s007.docx]

**S1 Table. TB-Stigma and depression symptoms**

| **TB-Stigma** | **No Depression**  **(n=358)** | **Major Depression syndrome**  **(n=39)** | | | **Other depressive syndrome**  **(n=47)** | | |
| --- | --- | --- | --- | --- | --- | --- | --- |
|  | **mean (SD)** | **mean (SD)** | **OR; 95%CI** | **aOR; 95%CI^a^** | **mean (SD)** | **OR; 95%CI** | **aOR; 95%CI^a^** |
| ***Patient perspective*** | |  |  |  |  |  |  |
| Total score | 17.50 (6.48) | 26.50 (7.89) | 1.21; 1.14-1.28 | 1.21; 1.13-1.29 | 21.53 (7.20) | 1.06; 1.01-1.11 | 1.07; 1.02-1.12 |
| Disclosure | 18.68 (9.11) | 29.38 (11.15) | 1.11; 1.07-1.15 | 1.10; 1.06-1.15 | 22.87 (10.46) | 1.03; 1.00-1.06 | 1.03; 1.00-1.07 |
| Isolation | 14.56 (7.40) | 22.86 (9.74) | 1.11; 1.06-1.15 | 1.11; 1.06-1.16 | 18.88 (9.35) | 1.04; 1.01-1.08 | 1.05; 1.01-1.09 |
| Guilty | 19.86 (9.04) | 27.49 (9.19) | 1.09; 1.05-1.13 | 1.08; 1.04-1.13 | 23.29 (8.40) | 1.03; 0.99-1.06 | 1.03; 0.99-1.06 |
| ***Community perspective*** | |  |  |  |  |  |  |
| Total score | 21.69 (7.77) | 28.93 (9.08) | 1.09; 1.05-1.13 | 1.10; 1.05-1.14 | 25.04 (7.63) | 1.03; 0.99-1.06 | 1.03; 0.99-1.07 |
| Isolation | 24.06 (8.36) | 32.34 (9.29) | 1.11; 1.06-1.16 | 1.10; 1.06-1.15 | 28.37 (7.42) | 1.04; 1.00-1.08 | 1.04; 1.00-1.08 |
| Distancing | 18.12 (9.02) | 23.82 (10.69) | 1.04; 1.01-1.08 | 1.05; 1.01-1.09 | 20.04 (10.98) | 1.00; 0.98-1.03 | 1.01; 0.98-1.04 |

^a^Adjusted with age, sex (male/female), group (intensive phase at public facilities/private facilities/LTFU to treatment/retreatment), area (urban/rural), job loss due to TB (yes/no), and formal education (no school/elementary/high school/university).
