# Supplementary material for: Stigma, depression, quality of life, and the need for psychosocial support among people with tuberculosis in Indonesia: A multi-site cross-sectional study
Source: PLOS Glob Public Health. 2024 Jan 8;4(1):e0002489. doi: 10.1371/journal.pgph.0002489 (PMC10773931; doi:10.1371/journal.pgph.0002489)
Supplement: S2 Table — (DOCX) [file pgph.0002489.s008.docx]

**S2 Table. TB-Stigma, depression, and quality of life between groups**

| **Outcome variables** | | **Group** | | | | | | | |
| --- | --- | --- | --- | --- | --- | --- | --- | --- | --- |
|  |  | **A: Treatment at public facility** | | **B: Treatment at private facilities** | | **C: LTFU to TB treatment** | | **D. Retreatment** | |
|  |  | *n=* | *404* | *n=* | *103* | *n=* | *51* | *n=* | *54* |
| TB Stigma, patient perspective | |  |  |  |  |  |  |  |  |
|  | No stigma | 51 | (13%) | 11 | (11%) | 8 | (16%) | 8 | (15%) |
|  | Low | 109 | (27%) | 23 | (22%) | 7 | (14%) | 14 | (26%) |
|  | Moderate | 238 | (59%) | 68 | (66%) | 33 | (65%) | 32 | (59%) |
|  | High | 6 | (1%) | 1 | (1%) | 3 | (6%) | 0 | (0%) |
|  | TB Stigma score, *median (min-max)* | *19.7 (0.0-50.0)* | | *21.2 (4.6-39.4)* | | *21.2 (0.0-36.4)* | | *18.2 (1.5-33.3)* | |
| TB Stigma, community perspective | |  |  |  |  |  |  |  |  |
|  | No stigma | 71 | (18%) | 18 | (17%) | 11 | (22%) | 13 | (24%) |
|  | Low | 42 | (10%) | 6 | (6%) | 5 | (10%) | 6 | (11%) |
|  | Moderate | 264 | (65%) | 73 | (71%) | 22 | (43%) | 32 | (59%) |
|  | High | 27 | (7%) | 6 | (6%) | 13 | (25%) | 3 | (6%) |
|  | TB Stigma score, *median (min-max)* | *23.3 (0.0-50.0)* | | *23.3 (6.7-45.0)* | | *28.3 (0.0-45.0)* | | *22.5 (0.0-46.7)* | |
| Depression symptom | |  |  |  |  |  |  |  |  |
|  | None | 231 | (57%) | 65 | (63%) | 30 | (59%) | 32 | (59%) |
|  | Mild | 112 | (28%) | 26 | (25%) | 15 | (29%) | 18 | (33%) |
|  | Moderate | 38 | (9%) | 5 | (5%) | 4 | (8%) | 2 | (4%) |
|  | Moderately severe | 17 | (4%) | 6 | (6%) | 1 | (2%) | 2 | (4%) |
|  | Severe | 6 | (1%) | 1 | (1%) | 1 | (2%) | 0 | (0%) |
| Major Depression Disorder | |  |  |  |  |  |  |  |  |
|  | None | 376 | (93%) | 97 | (94%) | 48 | (94%) | 52 | (96%) |
|  | Major depressive disorder | 28 | (7%) | 6 | (6%) | 3 | (6%) | 2 | (4%) |
| Quality of Life, *median (min-max)* | | *0.91 (0.00-1.00)* | | *0.84 (0.04-1.00)* | | *1.00 (0.00-1.00)* | | *0.84 (0.04-1.00)* | |
