# Supplementary material for: Stigma, depression, quality of life, and the need for psychosocial support among people with tuberculosis in Indonesia: A multi-site cross-sectional study
Source: PLOS Glob Public Health. 2024 Jan 8;4(1):e0002489. doi: 10.1371/journal.pgph.0002489 (PMC10773931; doi:10.1371/journal.pgph.0002489)
Supplement: S3 Table — (DOCX) [file pgph.0002489.s009.docx]

**S3 Table. Unmet need of psychosocial support and TB-Stigma**

| **Unmet need of psychosocial support** | **No**  **TB-Stigma**  **(N=78)** | | **Low**  **TB-Stigma**  **(N=153)** | | | **Moderate**  **TB-Stigma (N=371)** | | **High**  **TB-Stigma**  **(N=10)** | |
| --- | --- | --- | --- | --- | --- | --- | --- | --- | --- |
|  | **n** | **(%)** | **n** | **(%)** | **n** | | **(%)** | **n** | **(%)** |
| *Informational support* |  |  |  |  |  | |  |  |  |
| TB information for myself by HCW | 8 | (10.3) | 15 | (9.8) | 31 | | (8.4) | 2 | (20.0) |
| TB information for my family members by HCW | 10 | (12.8) | 20 | (13.1) | 59 | | (15.9) | 2 | (20.0) |
| TB information in a peer group meeting | 29 | (37.2) | 50 | (32.7) | 119 | | (32.1) | 6 | (60.0) |
| *Emotional support* |  |  |  |  |  | |  |  |  |
| Emotional support from HCW | 4 | (5.1) | 11 | (7.2) | 22 | | (5.9) | 1 | (10.0) |
| Emotional support from family | 0 | (0.0) | 7 | (4.6) | 11 | | (3.0) | 0 | (0.0) |
| Emotional support from peer | 20 | (25.6) | 47 | (30.7) | 100 | | (27.0) | 5 | (50.0) |
| *Instrumental support* |  |  |  |  |  | |  |  |  |
| Home visit by HCW | 17 | (21.8) | 31 | (20.3) | 85 | | (22.9) | 2 | (20.0) |
| Peer group meeting | 24 | (30.8) | 44 | (28.8) | 107 | | (28.8) | 6 | (60.0) |
| Individual counselling | 23 | (29.5) | 39 | (25.5) | 105 | | (28.3) | 5 | (50.0) |
| Group counselling | 24 | (30.8) | 42 | (27.5) | 107 | | (28.8) | 6 | (60.0) |
