# Supplementary material for: Stigma, depression, quality of life, and the need for psychosocial support among people with tuberculosis in Indonesia: A multi-site cross-sectional study
Source: PLOS Glob Public Health. 2024 Jan 8;4(1):e0002489. doi: 10.1371/journal.pgph.0002489 (PMC10773931; doi:10.1371/journal.pgph.0002489)
Supplement: Attachment — Submitted filename: TB stigma depression qol response.docx [file pgph.0002489.s010.docx]

Dr Tom Wingfield MbChB(Hons) PhD DTMH FRCP DipHIV DELE

Consultant Physician and Senior Clinical Lecturer

Departments of Clinical Sciences and International Public Health

Liverpool School of Tropical Medicine

Email: tom.wingfield@lstmed.ac.uk

Sunday 24^th^ September 2023

**Re: Response to editorial and reviewer comments on original research manuscript PGPH-D-23-00810 “Stigma, depression, quality of life, and the need for psychosocial support among people with tuberculosis in Indonesia: a multi-site cross-sectional study".**

Dear Dr Anil Gumber and PLOS Global Public Health Editorial Team,

We thank you, the editorial team and reviewers of PLOS Global Public Health for your encouraging, positive, and constructive review of our original research manuscript.

We are grateful that the reviewers commented that the research was important, well structured and written, well designed with appropriate methodology to answer the research questions, and with important findings that can be applied to developing a psychosocial intervention for TB-affected households in Indonesia.

We have revised and updated our manuscript accordingly, responding comprehensively to all Editor and Reviewer comments. In addition, we have included a completed copy of PLOS’ questionnaire on inclusivity and note the opportunity to submit future articles to the special edition on Global Mental Health with thanks.

**Reviewer #1:**

1. In the introduction, make sure that the objective and research question is clearly presented.

Response:

Thank you for this comment. We have revised the final paragraph of the introduction accordingly (lines 123-133): “For Indonesia to work towards ending TB, it will be essential to measure the “psychosocial” impact and consequences of TB such as TB-Stigma, depression, and QoL; and to identify and address the need for psychosocial support amongst TB-affected people. This much-needed data will support generation of evidence to inform the design and delivery of impactful, sustainable, and locally appropriate psychosocial interventions to reduce TB-Stigma, mitigate mental illness, and improve people with TB’s QoL. As part of a larger program of research, this study aimed to fill this knowledge gap in Indonesia by assessing TB-Stigma, depression, QoL and their correlations, as well as measuring the need for psychosocial support among adults with TB in Indonesia. The results of this study will support design of a community-based psychosocial support intervention and influence related national and international TB-Stigma policy and practice.”

2. Author should use sub-headings for the tools that were used to measure stigma, depression, QoL, etc.

Response:

Thank you for your suggestion. We have added subheadings for tools in the “Variables and instruments” section of the Methods, including “TB Stigma Scale”; “Patient Health Questionnaire-9 (PHQ-9)”; and “EQ-5D-5L”

3. Report the reliability and internal consistencies of the tools in this study.

Response:

We added the reliability and internal consistencies of the tools we used and also a lead-in sentence to the relevant paragraphs:

“As described further below, the tools used to measure TB stigma, depression, and QoL have all been previously validated for reliability and internal consistency in the Indonesian context.” (lines 192-195)

- TB Stigma Scale, “The tool was reliable— with a Cronbach's alpha of 0.738 for Form A (Patient Perspective) and 0.807 for Form B (Community Perspective).” (lines 201-202)
- PHQ-9, “We used the Indonesian-validated version of the Patient Health Questionnaire-9 (PHQ-9)(41), with a Cronbach's alpha of 0.837.” (lines 215-216)
- EQ5D5L, “This tool has been shown in other studies to be reliable with a Gwet’s test and restest agreement coefficient of 0.85–0.99 and percentage agreement of 90–99%.” (lines 228-230)
- Psychosocial support questions were validated prior to the implementation of our study. “The study team designed the initial questions related to needs and unmet needs for psychosocial support. Then, questions were shared with an expert panel including a pulmonologist, a psychologist, community medicine specialist, a national TB program staff, a member of TB civil society group to evaluated for content validity, and iteratively refined accordingly.” (lines 235-239)

4. Outcome and primary exposure covariates needed to be clearly mentioned in the method section separately.

Response:

Thank you for your feedback. In the original submitted manuscript, we included description of the covariates in the data analysis section. For clarity, and as suggested by the reviewer, we moved this sentence to lines 249-252, “We included several factors demonstrated in other studies (7, 8) in the analysis, which were age, sex (male/female), participant group (A, B, C, D), area (urban/rural), job loss due to TB (yes/no), and formal education level (no education/ elementary/high school/university).”

We also specified more clearly the co-primary outcome variables in the “Variables and instruments” section of the manuscript: “The key co-primary outcome variables informing estimates of psychosocial impact were TB-Stigma, symptoms of depression, QoL, and receipt of and/or unmet need for psychosocial support.” (lines 191-193)

5. Author should use the STOBE reporting guideline for cross sectional study.

Response:

A fully completed STROBE checklist is now attached as supplementary material.

6. Require a section on how the questionnaire was created and on which page what kind of information was collected. How the questionnaire was validated and piloted.

Response:

The full questionnaire consisted from four different section.

a. TB Stigma Scale, that we culturally adapted and validated from Van Rie’s original TB Stigma Scale. “We first created, culturally adapted, and validated an Indonesian version of the TB-Stigma Scale.(38) The Scale consisted of two forms: Form A (Patient Perspective) with 11 questions across three domains (disclosure, isolation, and guilty); and Form B (Community Perspective) with 10 questions across two domains (isolation and distancing). The tool was reliable— with a Cronbach's alpha of 0.738 for Form A (Patient Perspective) and 0.807 for Form B (Community Perspective).(38)” (lines 197-202)
b. The PHQ-9. We did not create the PHQ-9 questionnaire but it has been validated in Bahasa language in Indonesia in other studies, which we reference. “We used the Indonesian-validated version of the Patient Health Questionnaire-9 (PHQ-9)(41), with a Cronbach's alpha of 0.837,(38) to assess the participants’ symptoms of depression according to the pre-existing PHQ-9 categories: no depression (score of 0-4), mild (5-9), moderate (10-14), moderately severe (15-19), and severe (20-27) [41].” (lines 215-218)
c. EQ-5D-5L is a standard questionnaire developed by EuroQOL. QoL was measured using the Indonesian version of EQ-5D-5L(42, 43) consisting of five locally-weighted dimensions: mobility, self-care, usual activities, pain/discomfort, and anxiety/depression with five levels of severity.(42) This tool has been shown to be reliable in other studies with a Gwet’s test and retest agreement coefficient of 0.85–0.99 and percentage agreement of 90–99%.(42)” (lines 226-230)
7. “Ten questions were added to measure participants’ receipt of and/or perceived unmet need for psychosocial support.” Was it a valid tool like PHQ-9? If not, how was this tool validated? Internal consistencies require reporting.
   In the statistical analysis, the raised research question in the introduction should be answered.

Response:

One of the key aims of this study was to measure the need for psychosocial support among adults with TB in Indonesia. We answered this objective through the analysis summarized in Table 6. Specifically, we measured the receipt of, the need for, and the unmet need for psychosocial support.

As appropriate for this “unmet need” questionnaire, which is not a scale, the questions were validated for content. Such content validation is appropriate to ensure that the items reflected the needs of psychosocial support among people with TB based on three recognized theoretical domains.

We have described this more clearly in the updated manuscript as follows:

“The study team designed the initial questions related to needs and unmet needs for psychosocial support. Then, questions were shared with an expert panel including a pulmonologist, a psychologist, community medicine specialist, national TB program staff, and a member of a TB civil society organization. The panel evaluated the questions for content validity and iteratively refined the questions. Questions were divided into three recognized domains: informational, emotional, and instrumental support.(44) Informational support refers to any facts, advice, or other educational materials provided to people with TB to help them solve problems. Emotional support refers to care, encouragement, and empathy to garner a sense of security amongst TB-affected people. Informational support in this study was defined as a tangible help in practical form provided through material assistance or practical program, such as counseling or meeting. (lines 231-239)

8. Was the assumption of the models met? Authors should report all the assumptions of all models that they used and how the model was built, such as variable selection, check of correction, interaction/effect modification, and confounders.

Response:

As per our response to Reviewer 1’s Comment 4 above, we included the relevant covariates known to be associated with depression and QoL based on previous evidence (see references 7 and 8, lines 250). These variables were treated as interaction variables, while TB Stigma Scores were the key outcome variable with the main effect in the model.

9. The author can not include the variables in the model without forming research questions that they are looking to answer, and variable selection should be explained with the check of correction, interaction/effect modification, and confounders.

Response:

Thank you for the comment. We added a sentence to explain this, “After reviewing the literature, with special reference to the conceptual framework in Chen et al [19], we designed the model to evaluate at the interaction between TB Stigma, depression, and QoL.” (lines 274-275) Coupled with our response to Reviewer 1’s Comment 8 above, we are confident that the research questions and variable selection for the model is now clear in the updated manuscript.

10. All models should be reported their fitness.

Response:

Thank you for the suggestion. We added the model fitness accordingly.

- Factors related to TB Stigma Scores: “The models were fit (R-Square= 0.066, F=4.238, p<0.001 for patient perspective; R-square= 0.046, F=2.887, p=0.002 for community perspective).” (lines 334-336)
- TB Score and depression: “The model assessing correlation between TB Stigma Scores and depression, with adjustment for age, sex, Group, rural vs urban area, and job loss due to TB, were fit (χ^2^=96.926, p<0.001 for TB Stigma patient perspective; χ^2^=66.670, p<0.001 for TB Stigma community perspective).” (lines 346-348)
- TB Score, depression, and QoL: “The model assessing correlation between TB Stigma, depression, and QoL with adjustment for age, sex, Group, rural vs urban area, and job loss due to TB, were fit (R-square= 0.324, F=23.878, p<0.001 for TB Stigma patient perspective; R-square= 0.104, F=6.350, p<0.001 for TB Stigma community perspective).” (lines 359-361)

11. Results should be presented in sub-headings that will portray the research questions that they are going to answer.

Response:

Thank you very much for your suggestion. We added subheadings accordingly.

**Reviewer #2:**

This is an important work on stigma, depression and quality of life of people with drug sensitive tuberculosis in Indonesia. The research is well designed with the appropriate methodology to respond to the research questions. Overall, the findings are important to design appropriate psychosocial support for the patients.
The overall structure of the manuscript is very good with clear flow of each part to tell the story. I have the following comments:

1. In the methods part the stigma was classified as no stigma, low, moderate and high based on the quartile of the cohort. I believe this misleading, without the presences of a validated cutoff point, to divided stigma in such a way is not helpful. Even, a single experience in one of the question included in the scale might have profound impact on the patients outcome. I would suggest the authors treat stigma as a continues variable and present the mean and standard deviation across the manuscript. Accordingly the statement '60.6% experienced moderate TB-Stigma' in the abstract should be revised, please included the mean +/- SD and the range.

Response:

Thank you for your comment and suggestion. We agree and, as the reviewer has recommended, did treat the TB Stigma as a continuous variable (lines 311-312 and 319-320). However, while displaying the score as a continuous variable is strongest from a statistical perspective, it may not be practical for readers – especially policy makers in this field - to interpret. For example, how is a score of 17.35 interpreted and contextualised and what future policy should be applied? Therefore, we tried to modify the score into low, medium, and high TB-stigma levels to enhance interpretation and facilitate practical policy dialogue. There is existing evidence and support for this approach, which is well used and documented in the related TB-stigma literature including the Stop-TB Stigma Measurement Handbook. For these reasons, we have reported TB-stigma both as a continuous variable and in categories. However, we agree that the cut-off should be validated and we added it as one of limitations of this study (line 480-483).

2. In the abstract 'depression was identified in 41.5% participants', you provided the point estimates. It would be good to included the 95%CI.

Response:

Thank you for your suggestion. We added it accordingly, “Depression was identified in 41.5% (95% CI 37.7-45.3%) participants.” (line 71)

We also added the 95% CIs in the main text. (line 339)

3. How Patient Health Questionnaire-9 (PHQ-9), results are interpreted is not included in the main manuscript, say what is mild or moderately severe depression means. The authors should also provide references to the sources of such classifications.

Response:

Thank you for your suggestion. We added the interpretation of PHQ-9 scores and the relevant well-cited reference accordingly, “the pre-existing PHQ-9 categories: no depression (score of 0-4), mild (5-9), moderate (10-14), moderately severe (15-19), and severe (20-27).” (lines 218-219)

4. When presenting the mean for the different tool for the first time, it is important to indicate the range (minimum and maximum scores). This will help to put the results into context.

Response:

Thank you for your suggestion. We revised them accordingly.

- “The average TB-Stigma score in Form B (Community Perspective) was 23.4 (SD 8.4, min-max 0-50). Most participants (63.9%) reported moderate perceived TB-Stigma from their community, particularly the perception of being isolated from and by people in their community (38.2-66.7%).” (lines 320-323)
- “The average QoL among adults with TB without depression was 0.9 (SD 0.13, min-max 0.28-1.00).” (lines 357-358)

**Journal requirement**

1. Please include a complete copy of PLOS’ questionnaire on inclusivity in global research in your revised manuscript. Our policy for research in this area aims to improve transparency in the reporting of research performed outside of researchers’ own country or community. The policy applies to researchers who have travelled to a different country to conduct research, research with Indigenous populations or their lands, and research on cultural artefacts. The questionnaire can also be requested at the journal’s discretion for any other submissions, even if these conditions are not met. Please find more information on the policy and a link to download a blank copy of the questionnaire here: https://journals.plos.org/globalpublichealth/s/best-practices-in-research-reporting. Please upload a completed version of your questionnaire as Supporting Information when you resubmit your manuscript.”

Response:

We filled and thereby attached the required PLOS’ questionnaire on inclusivity in global research.

2. Please provide separate figure files in .tif or .eps format.

Response:

We uploaded the figure separately in .tiff format.

**Additional Editor Comments (if provided):**

Some additional Analysis is required by Four Groups of Patients. Most tables including depression outcomes need to be tabulated by Four Groups. I am surprised the data on EQ5D is collected but analysis is not done for each of five dimensions across four groups. Also I am unable to see analysis based on QALYs score.

Response:

Thank you very much for your suggestion. We added the tabulation by four groups in Supplement T1, and added a sentence in the main text, “While TB Stigma scores among those LTFU and those receiving treatment at private facilities were higher than those treated at public facilities, their QoL was significantly lower. (**Supplement T1 and F2**).” (lines 367-369) In response to QALY, we analysed the quality of life—based on utility score—and did not proceed with the QALY. This study did not intend to measure QALYs and, therefore, did not follow the calculation of utility score with the life years. We hope to include QALY calculation in follow-on research we are currently conducting, in which we are implementing and evaluating a psychosocial support intervention for TB-affected households in Indonesia.

We are grateful to the editorial team and reviewers for their excellent comments, which have helped to make the revised manuscript stronger than the original. We are confident that the updated and improved manuscript is now ready for further consideration for publication in PLOS Global Public Health.

Yours sincerely,

Dr T Wingfield (corresponding author on behalf of the team)
